# Two rare cases of a solid pseudopapillary neoplasm of the pancreas

**DOI:** 10.3892/ol.2013.1476

**Published:** 2013-07-19

**Authors:** CHIEMI SAIGO, YOSHINOBU HIROSE, NAMI ASANO, MANABU TAKAMATSU, NORIYOSHI FUKUSHIMA, ICHIRO YASUDA, SATOSHI GOSHIMA, MICHIO OZEKI, SHINJI OSADA

**Affiliations:** 1 Department of Pathology, Gifu University Hospital, Gifu 501-1194, Japan; 2 Department of Tumor Pathology, Gifu University Graduate School of Medicine, Gifu 501-1194, Japan; 3 Department of Pathology, Jichi Medical University Hospital, Shimotsuke, Tochigi 329-0498, Japan; 4 Department of Internal Medicine, Gifu University Graduate School of Medicine, Gifu 501-1194, Japan; 5 Department of Radiology, Gifu University Graduate School of Medicine, Gifu 501-1194, Japan; 6 Department of Pediatrics, Gifu University Graduate School of Medicine, Gifu 501-1194, Japan; 7 Department of Surgical Oncology, Gifu University Graduate School of Medicine, Gifu 501-1194, Japan

**Keywords:** solid-pseudopapillary neoplasm, cytology, CD10, β-catenin, trypsin

## Abstract

A solid pseudopapillary neoplasm (SPN) of the pancreas has distinct histopathological features. A solid pattern of growth with pseudopapillary structures that result from degeneration is observed. On rare occasions, the tumor may vary from being entirely solid to completely cystic. The present study describes two unique cases of SPN. A 25-year-old male presented with a pancreatic tumor showing a predominantly solid pattern with no degenerative change, although the pre-operative cytological specimens that were obtained by endoscopic ultrasound-guided fine-needle aspiration (EUS-FNA) revealed pseudopapillary structures. The second case was of an 11-year-old female who presented with a pancreatic tumor with prominent degeneration. Nests and cords of the remaining neoplastic cells were located only at the periphery, with perineural invasion. An immunohistochemical analysis revealed that the tumor cells in the two cases were positive for CD10 and β-catenin and negative for trypsin. An awareness of the broad morphological variability of SPN and an immunohistochemical panel that includes CD10, β-catenin and trypsin are useful for establishing an accurate diagnosis.

## Introduction

A solid pseudopapillary neoplasm (SPN) is a low malignant epithelial tumor of the pancreas that predominantly occurs in adolescent and young females (1,2). In unusual presentations, SPN may occur in older patients, ectopic locations and males. Histopathologically, the tumor has distinct features; a solid pattern of growth with the semblance of papillary structures that result from degeneration are observed. However, there is a broad variability of the morphology of SPN. Certain tumors have a bloody appearance with only scattered tumor foci, while others may be solid and fleshy throughout (3). These morphological variations in the characteristics of the tumors represent a diagnostic challenge for pathologists and clinicians. The present study describes two rare cases of SPN, one with an extremely solid pattern and one with an almost degenerative appearance, and discusses how to establish an accurate diagnosis in unusual cases. Written informed consent was obtained from the patients.

## Case reports

### Case 1

**Figure 1:**
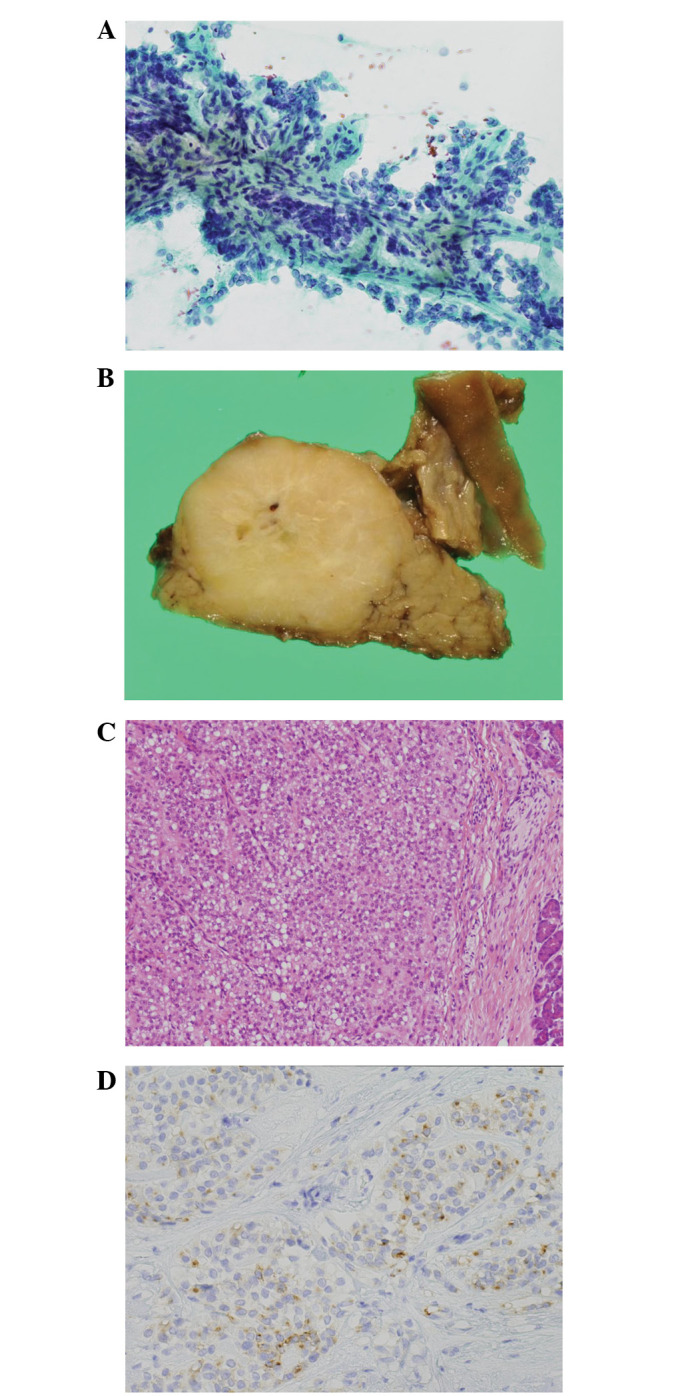
SPN of case 1. (A) Cytological examination demonstrating papillary-like structures (magnification, ×200). (B) Gross examination exhibiting a solid tumor with no cystic change. (C) The tumor showing an entirely solid pattern (HE staining; magnification, ×100). (D) Immunostaining of synaptophysin revealing focal, dot-like positivity (magnification, ×200). SPN, solid papillary neoplasm; HE, hematoxylin and eosin.

A 25-year-old male was admitted to Gifu University Hospital (Gifu, Japan) with a two-week history of diarrhea and abdominal pain. The results of routine laboratory tests were all within the normal range. However, an abdominal ultrasound revealed a mass in the pancreatic head, which was composed of high-echoic central and low-echoic peripheral areas. Computed tomography (CT) and magnetic resonance imaging identified the nodule to be predominantly solid and ~28 mm in maximal diameter. The cytological smear and cell block of the sample that was obtained by endoscopic ultrasound-guided fine-needle aspiration (EUS-FNA) revealed the presence of uniformly monomorphic tumor cells, several layers of which covered the central fibrovascular stalks and formed papillary-like structures (Fig. 1A). The patient underwent a pancreatoduodenectomy. The histological examination demonstrated that the tumor exhibited a solid pattern as a whole with no cystic change, comprising of sheets and cords of oval-to-round monomorphic cells with cytoplasmic vacuolization in certain areas (Fig. 1B and C). A fibrous area was observed at the center of the tumor, which may have been generated by the preceding needle aspiration. An immunohistochemical analysis showed that the tumor cells were positive for neuron specific enolase (NSE), CD56, chromogranin, synaptophysin, β-catenin and CD10 and negative for trypsin (Fig. 1D).

### Case 2

**Figure 2:**
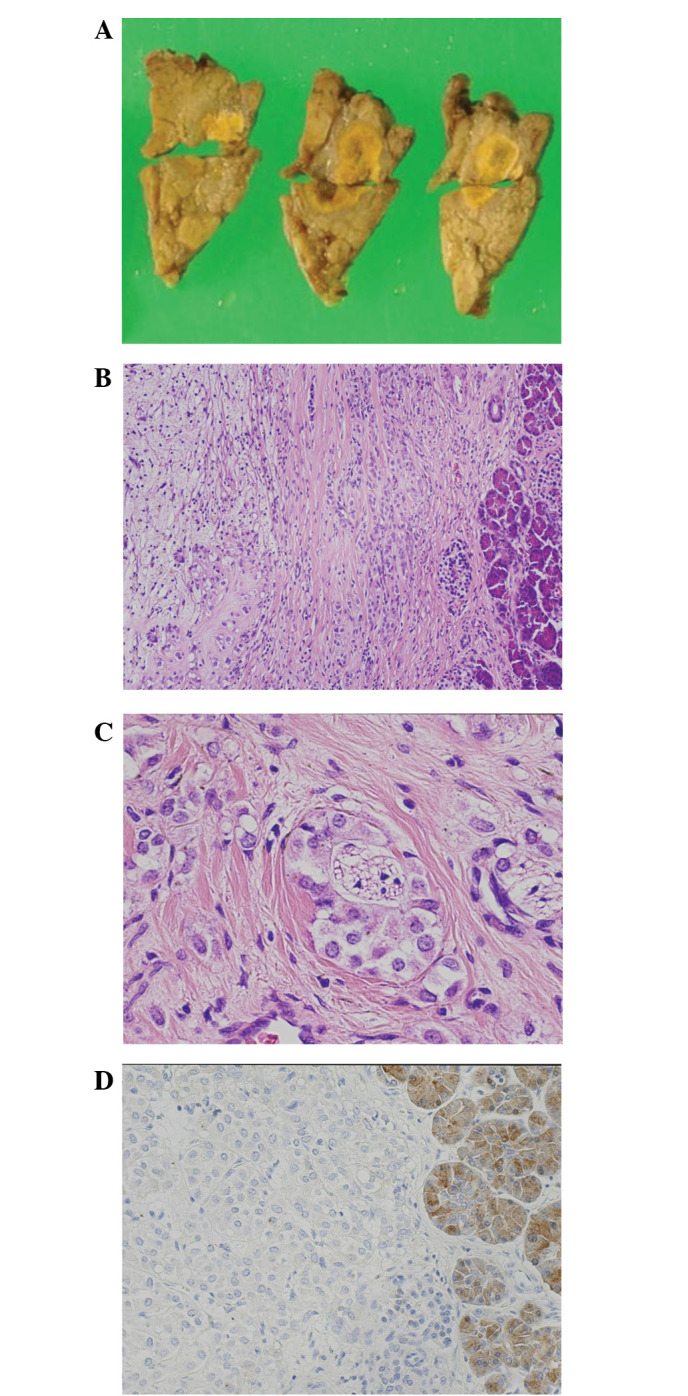
SPN of case 2. (A) The gross appearance of the mass is yellowish with hemorrhagic degeneration. (B) The tumor has central degeneration with a cellular component at the periphery (HE staining; magnification, ×100). (C) Perineural invasion in the tumor (HE staining; magnification, ×400). (D) Immunostaining of trypsin revealing no positivity (magnification, ×200). SPN, solid papillary neoplasm; HE, hematoxylin and eosin.

An 11-year-old female presented to Gifu University Hospital due to a sudden onset of abdominal pain. Laboratory tests revealed a high level of serum amylase, suggesting acute pancreatitis. An abdominal CT scan disclosed a cystic mass (35 mm in maximal diameter) with a clear margin in the pancreatic tail. The follow-up examination revealed that the size of the mass had become smaller within the next three months with no treatment, attaining a size of 18 mm in maximal diameter. The lesion was excised by a distal pancreatomy. Prior to the surgery, no histological or cytological examinations were performed. On gross examination, the yellowish tumor was prominently degenerative with cystic and hemorrhagic changes (Fig. 2A). Microscopically, the neoplastic solid areas consisted of cords and nests of monomorphic cells with oval-to-round nuclei, which were located only at the periphery (Fig. 2B). The remaining tumor area appeared to be solid with no pseudopapillary change and possessing perineural invasion (Fig. 2C). The immunohistochemistry analysis revealed that the tumor cells were positive for NSE, CD56, β-catenin and CD10 and negative for chromogranin, synaptophysin and trypsin (Fig. 2D).

In cases 1 and 2, a diagnosis of SPN was confirmed and no adjuvant therapy was administered. No residual tumor or metastases were identified during the follow-up period.

## Discussion

The microscopic appearance of an SPN is variable (4,5). Histopathologically, the neoplasm has distinct features in the majority of cases, consisting of solid areas alternating with pseudopapillary formations. However, the contribution of each component varies greatly as ~10% of SPNs are either entirely solid or completely cystic (1). The extremely solid pattern, as shown in case 1, may cause difficulty in distinguishing between an SPN and a neuroendocrine tumor (NET), since the histopathological presentation of the latter tumor is usually a solid pattern of growth. In order to differentiate between these two tumors, an awareness of the cytological features of SPNs, obtained by EUS-FNA, is beneficial to a great extent. In case 1, several cellular aggregates with papillary-like structures were confirmed on the cytological smear and cell block, present partly due to the artificial effect of EUS-FNA, which may have given rise to and/or emphasized the pseudopapillary change. In addition, the cytological examination revealed the cellular features of the SPN more clearly, depicting monomorphic cells with loose cohesiveness and scattered nuclear grooves. The observations in the present study are consistent with the suggestion by Bardales *et al* that the EUS-FNA diagnosis of an SPN is considered to be accurate (6). Pettinato *et al* also suggested that a cytological diagnosis of SPN may be rendered with great confidence in unusual presentations, including those that are identified in older patients, males, ectopic locations and metastatic sites (7).

The evaluation of neuroendocrine differentiation in the tumors using immunohistochemical markers, including synaptophysin, chromogranin, CD56 and NSE, is also important in order to differentiate between an SPN and an NET. Caution should be paid, however, since the cellular differentiation of SPNs remains to be elucidated and, therefore, immunohistochemistry may be of marginal use in this context (8). In general, the neoplastic cells of an NET are immunopositive for all the neuroendocrine markers, while the tumor cells of an SPN are positive for NSE and CD56 and mostly negative for synaptophysin and chromogranin. However, it is noteworthy that a small number of SPNs express synaptophysin and chromogranin, although weakly and focally (8–10). The tumor cells in case 1 were immunopositive for synaptophysin and chromogranin in a scattered fashion and for NSE and CD56 in a diffuse manner.

In certain instances, SPNs are prominently cystic, with only thin peripheral rims of the remaining tumor cells, as in case 2. Notably, perineural invasion was identified in the remaining tumor area of case 2, which may indicate malignant behavior (2). Hence, acinar cell carcinoma (ACC) should be included in the differential diagnosis of this case, as rare findings of gross necrosis and degenerative cystic changes exist in this malignant tumor (11). To differentiate an SPN from an ACC, it is important to identify acinar cell differentiation in the tumor tissue. To do so, the immunohistochemical analysis of the expression of pancreatic enzymes, including trypsin, chymotrypsin and lipase, is necessary. Among these enzymes, trypsin is immunohistochemically detectable in >95% of ACC cases, and has been regarded as the most diagnostically useful marker (12,13). In case 2, the immunohistochemical reactivity of trypsin was not present in the tumor, suggesting that it did not have apparent acinar cell differentiation and, consequently, was not diagnostic of ACC. The immunohistochemical analysis did not examine α1-antitrypsin in either of the cases, as this serine protease inhibitor is a non-specific marker for acinar cell differentiation, whose physiological target is leukocyte elastase rather than trypsin (12).

Studies have shown that immunohistochemical CD10 and β-catenin are valuable to establish a diagnosis of SPN. CD10 is a cell-surface neutral endopeptidase, an antibody which is used in daily practice as a marker of several cell lineages, including germinal center cells, renal tubular or glomerular cells and endometrial stromal cells (14). In a study by Notohara *et al*, it was reported that CD10 immunopositivity was detected diffusely in all the SPNs that were examined, whereas the immunopositivity was scattered in 67% of ACCs and focal in 20% of NETs (15). Furthermore, genetic events, including a mutation or truncation of the β-catenin gene, have been identified in 83% of SPNs, 23.5% of ACCs and 0% of NETs, suggesting that genetic alterations of β-catenin may play a role in the pathogenesis of certain pancreatic tumors (16,17). Immunohistochemistry analyses have revealed that the nuclear and cytoplasmic overexpression of β-catenin may be observed in 95% of SPNs, 5% of ACCs and 0% of NETs (13,17). These findings indicate that the immunostaining of CD10 and β-catenin may be useful as markers for SPN and that the immunohistochemical panel, including these new markers, is warranted for the differential diagnosis of SPN. Additionally, the diffuse positivity of CD10 and the nuclear and cytoplasmic staining of β-catenin in the two cases of the present study strongly favored the diagnosis of an SPN.

The reason why SPN has numerous morphological variations remains undetermined. A plausible explanation is the size of the tumor. The solid variants of SPN represent a number of small tumors that have not grown large enough to undergo cystic degeneration (18). Another assumption provided by Takahashi *et al* is that of a gender difference (8). The study reported that SPNs in male patients tended to be predominantly composed of solid components without degenerative changes in comparison with the female counterparts, although the neoplasms of the male patients that were examined were of a similar size to those observed in the females. Furthermore, genetic factors, including β-catenin, may be responsible for the morphological variables. β-catenin is a significant molecule in cell-cell adhesion and genetic aberrations of the gene may give rise to the detachment of adhesion by the reduction in the expression of E-cadherin (19). Therefore, it is possible that alterations in β-catenin may play a role in the disengagement between tumor cells, causing the cystic degenerative changes of SPNs. Further studies are required to clarify this mechanism.

In summary, the current study presents two cases of rare SPNs with unusual macroscopical and microscopical appearances. The literature suggests that when the solid or cystic area is predominant in an SPN, a detailed observation and careful interpretation of the cytological and immunohistochemical findings may be useful to avoid a potential misdiagnosis.
